# WISECONDOR: detection of fetal aberrations from shallow sequencing maternal plasma based on a within-sample comparison scheme

**DOI:** 10.1093/nar/gkt992

**Published:** 2013-10-28

**Authors:** Roy Straver, Erik A. Sistermans, Henne Holstege, Allerdien Visser, Cees B. M. Oudejans, Marcel J. T. Reinders

**Affiliations:** ^1^Delft Bioinformatics Lab, Delft University of Technology, Mekelweg 4, 2628 CD Delft, The Netherlands, ^2^Department of Clinical Genetics, VU University Medical Center Amsterdam, van der Boechorststraat 7 (BS7/J377), 1081 BT Amsterdam, The Netherlands and ^3^Department of Clinical Chemistry, VU University Medical Center Amsterdam, van der Boechorststraat 7 (BS7/J377), 1081 BT Amsterdam, The Netherlands

## Abstract

Genetic disorders can be detected by prenatal diagnosis using Chorionic Villus Sampling, but the 1:100 chance to result in miscarriage restricts the use to fetuses that are suspected to have an aberration. Detection of trisomy 21 cases noninvasively is now possible owing to the upswing of next-generation sequencing (NGS) because a small percentage of fetal DNA is present in maternal plasma. However, detecting other trisomies and smaller aberrations can only be realized using high-coverage NGS, making it too expensive for routine practice. We present a method, WISECONDOR (WIthin-SamplE COpy Number aberration DetectOR), which detects small aberrations using low-coverage NGS. The increased detection resolution was achieved by comparing read counts within the tested sample of each genomic region with regions on other chromosomes that behave similarly in control samples. This within-sample comparison avoids the need to re-sequence control samples. WISECONDOR correctly identified all T13, T18 and T21 cases while coverages were as low as 0.15–1.66. No false positives were identified. Moreover, WISECONDOR also identified smaller aberrations, down to 20 Mb, such as del(13)(q12.3q14.3), +i(12)(p10) and i(18)(q10). This shows that prevalent fetal copy number aberrations can be detected accurately and affordably by shallow sequencing maternal plasma. WISECONDOR is available at bioinformatics.tudelft.nl/wisecondor.

## INTRODUCTION

Classical methods for prenatal testing are karyotyping or quantitative fluorescence polymerase chain reaction, which require invasive chorionic villus sampling (CVS) or amniotic fluid sampling. Both procedures are associated with a 1:100 chance of consequent miscarriage (1).

Previous studies have shown that a small fraction of cell-free DNA in maternal plasma is of fetal origin (2). This fraction was found to range between 3.4% in early pregnancy and 6.2% in late pregnancy (3) and uniformly distributed over the whole genome (4). With the increasing quality of next-generation sequencing (NGS) data, this small fraction of fetal DNA has proved to be enough to detect fetal aberrations. Using NGS data of maternal plasma samples, Chiu *et al.* (5) developed a *z*-score method to detect pregnancies of children with trisomy 21, associated with Down syndrome. This method proved reliable for shallow sequenced samples (6,7) and is already applied in clinical settings. The downside of the method is that it requires re-sequencing of known healthy reference samples everytime a new set of samples is tested to minimize experimental influences on sequencing depth variations, thus increasing testing costs.

Although this method was designed to detect genetic aberrations in singleton pregnancies, Canick *et al.* (8) showed promising results of its application to detect trisomy 21 in twin pregnancies as well. Several other attempts were made to extend the method to the detection of trisomy 13, trisomy 18 (9) and gender classification. These classifications turned out to be more difficult because of chromosome-dependent sequencing biases. Jiang *et al.* (10) created a tool based on a student’s *t*-test rather than a *z*-score, which shows improved results in detection of trisomy 13, 18 and 21, as well as gender-specific trisomies. Among others, Sehnert *et al.* (11) stress the influence of ‘interrun’ and ‘intrarun’ sequencing variation. To overcome interrun variations, they propose normalization of the read counts on one chromosome with the total number of reads on a predefined set of chromosomes instead of the total number reads in the sample. The actual significance of chromosomal aberrations is then determined on the set of control samples, similar to Chiu *et al.* Although this provides an interesting improvement to solving interrun variations, several abnormalities are left undetected.

In an attempt to increase the resolution of detectable aberrations, one study shows results in detecting DiGeorge syndrome, a deletion of 3 Mb in 22q11.2 (12). They pointed out that there was a statistical difference in read depth of the targeted area between healthy samples and samples containing a deletion in this region. Although the two test samples used did result in *z*-scores <−3 (considered to be significant) for the targeted area, one of the 14 healthy samples had a *z*-score >3, making it difficult to assess clinical applicability. A study by Srinivasan *et al.* (13) showed that deep sequencing of maternal plasma (400–750 M reads per sample) allowed detection of several small fetal Copy Number Variations (CNVs). Read depths of bins as small as 100 kb were compared over several samples, reporting fetal aberrations down to 0.2 Mb in size. Yu *et al.* (14) used a similar approach to obtain diagnostic sensitivity indications for the required amount of reads for detection of fetal CNVs between 1 and 3 Mb in size, using 125–480 M reads per sample. For the detection of aneuploid cancer cells in the blood stream, Chan *et al.* (15) designed an approach to detect copy number aberrations in blood plasma that also makes use of binning. They suggest to correct for known GC biases using locally weighted scatterplot smoothing (LOWESS). However, relatively deep coverage was necessary to determine the significance of the aberrations based on a global statistical analysis of read-depths for all bins. At lower coverages the remaining variations over the bin positions is not taken into account by this approach.

Another study by Lo *et al.* (4) showed that it is possible to construct a genome-wide fetal map and determine the mutational status of the fetus from maternal plasma DNA using information about the paternal genotype and maternal haplotype. However, the paternal genotype and the maternal haplotype had to be determined separately and the maternal plasma containing fetal DNA was sequenced at 65-fold coverage, rendering this method too expensive for routine clinical application. Recent research shows that it is possible to determine almost the complete fetal genome correctly by integrating the haplotype-resolved genome sequence of the mother, the shotgun genome sequence of the father and the deep sequencing of cell-free DNA in maternal plasma (16). Although the results of this study are promising, obtaining the required amount of data is, again, far too expensive for routine clinical application. It would be appealing for clinical practice if similar results could be obtained with coverages close to or below 1×-fold to keep costs at the same level as current practice for trisomy detection, without the requirement of paternal information.

Recently, Chen *et al.*(17) presented an approach that corrects the read-depth signal using a GC correction and subsequently segments the signal. The resulting segments are compared with control samples to determine the significance of their aberration. This approach allowed the detection of aberrations down to 10 Mb within the four positive samples tested. The idea of a window-based correction for GC content is promising, but the segmentation scheme for detecting break points can be sensitive and problematic with larger aberrations. We adopt a similar window-based approach but avoid the need to segment the read-depth signal. For that we propose a novel scheme in which the aberrations are determined based on a within-sample comparison, thus not relying on control samples anymore. With that we realize a robust calling scheme that also can detect small aberrations in low coverage data.

The aim of this study was to determine whether T13, T18 and T21 as well as subchromosomal genetic disorders could be detected using ‘only’ a maternal plasma sample containing fetal DNA and shallow sequencing. Also, to decrease sequencing costs even further, we aimed to reduce the amount of required reference samples per sample test. The method that we propose is called WISECONDOR (WIthin-SamplE COpy Number aberration DetectOR) and is able to call fetal genetic disorders using shallow sequencing without the necessity to re-sequence healthy samples for normalization.

## MATERIALS AND METHODS

In an attempt to develop a high-resolution version of the method described by Chiu *et al.* (5), we applied and tested the following alterations. (i) We divided the whole Hg19 reference genome into bins and determined the read-depth for each bin. (ii) We determined the GC-content for each bin and fitted a LOWESS function (18) to the GC-count versus the read depth for each bin. (iii) The read depth for each bin was subsequently divided by the LOWESS value of its corresponding GC-content (Supplementary Figure S4), turning it into a GC-corrected read frequency. (iv) For each bin, we compared the normalized read frequencies to the normalized read frequencies in the same bins in a reference set of other normal (diploid) samples using a *z*-score method. This allowed us to detect most of the whole-chromosome copy number changes, but attempts to detect copy number changes at a higher resolution resulted in numerous false positives and false negatives (Supplementary Tables S1 and S2). Although this method corrects for local mapping biases, it is too sensitive to noise for small read frequency differences among samples.

Therefore, we decided to apply a ‘within-sample’ comparison using reference bins that have a similar behavior compared with the test bin. Different regions on the genome vary in read frequency characteristics from one sample to another. While between-sample comparison suffers from this, within-sample comparison does not: regions with equal characteristics will behave alike within a test sample, as all regions are subject to the same experimental procedures. This removes the need to fully understand the true reasons for read frequency variations over different samples.

**Figure 1.**
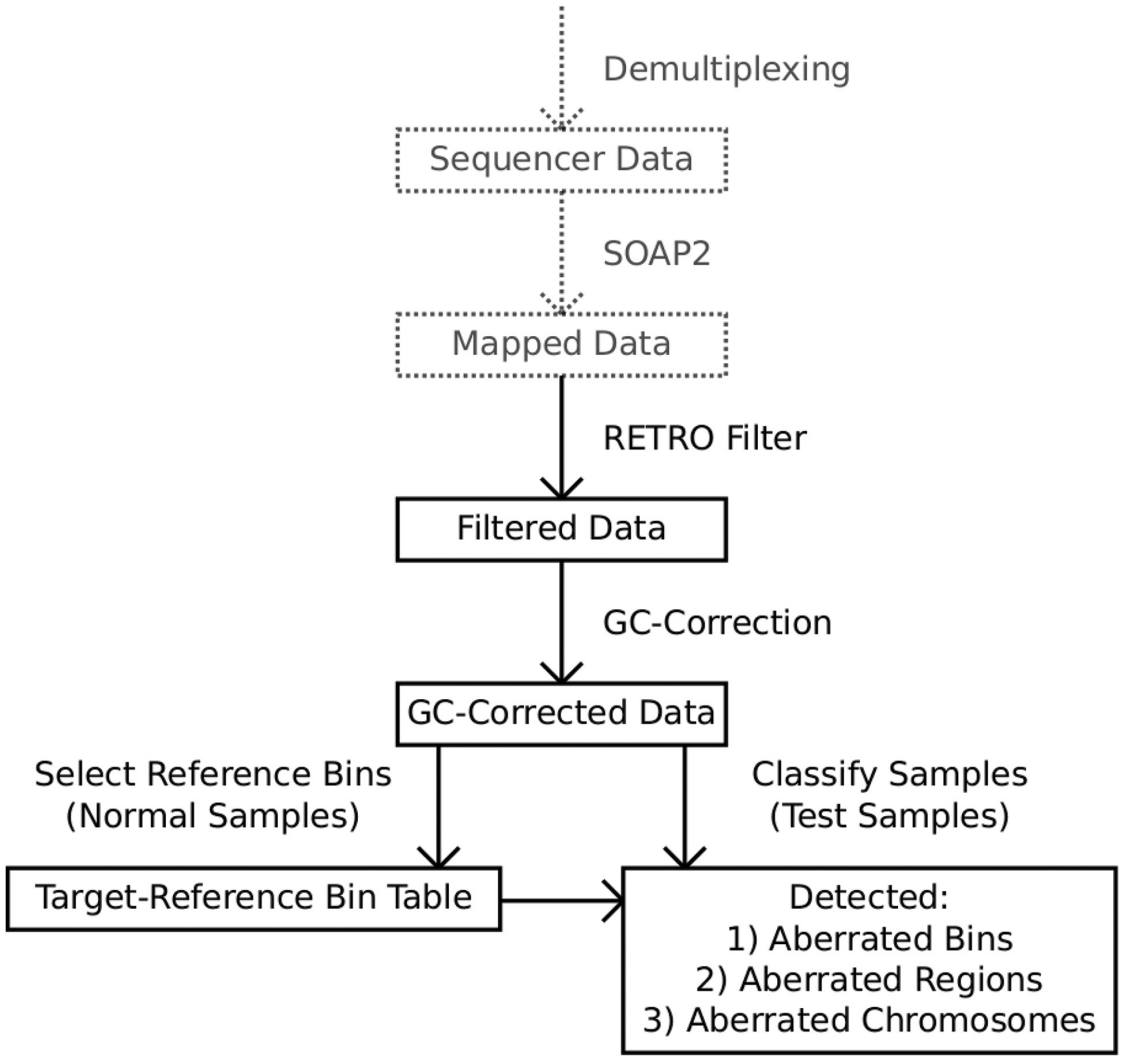
Overview of WISECONDOR, showing the data flow starting at the top through all main steps into the classification at the bottom. Note that ‘test samples’ and ‘reference samples’, used to find the reference bins, follow different paths in the data flow. The dashed steps are not part of WISECONDOR and can be interchanged with other mapping strategies, such as BWA (19), BowTie (20), etc.

**Figure 2.**
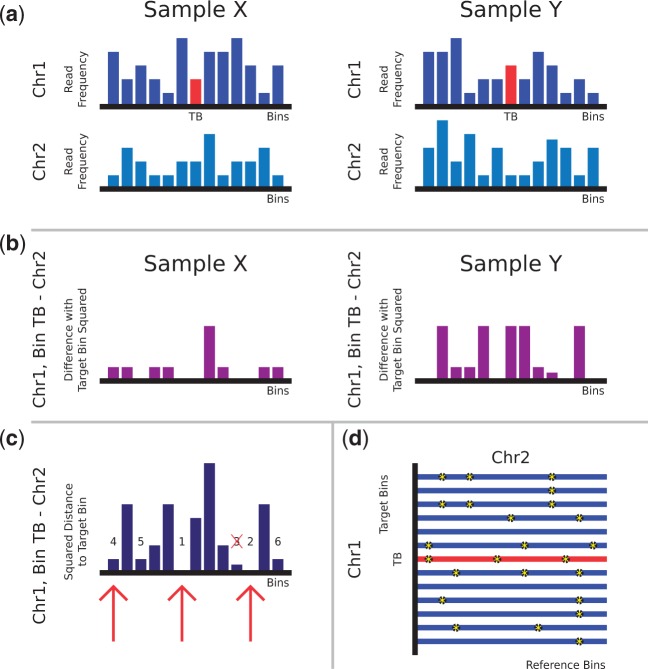
Finding within-sample reference bins by WISECONDOR. (**a**) Shows an area on chromosomes 1 and 2 for two normal (diploid) samples X and Y. The red bar is the target bin (*TB*) for which a set of reference bins is to be determined. Reference bins are not allowed to be present on the same chromosome. Hence, the set of reference bins for the indicated target bin in this example all need to be on chromosome 2. (**b**) Squared differences between target bin *TB* and each of the bins on chromosome 2 for both samples. (**c**) Summation of the squared differences between target bin *TB* and each of the bins on chromosome 2 over both samples. Numbers show the similarity ranking of the bins with respect to target bin *TB*. Red arrows indicate the bins chosen for target bin *TB* to be included in the set of reference bins. The bin ranked third is not included because it is directly connected to a bin previously selected (bin 2). (**d**) Stars on each row illustrate selected reference bins on chromosome 2 for every bin of chromosome 1. Notice that the set of reference bins differs for different target bins (rows in this panel).

This is implemented in a pipeline called WISECONDOR, which is able to call high-resolution fetal genetic disorders using shallow sequencing. Figure 1 shows an overview of the developed pipeline, while Figure 2 provides an illustration of how reference bins are determined, as described below. An overview of all WISECONDOR options is shown in Supplementary Table S3.

## WISECONDOR: within-sample reference bins

Although WISECONDOR uses a within-sample comparison, it still uses a set of normal diploid samples to identify the within-sample reference bins that behave similarly to the bin that is being tested (denoted as the target bin). The whole genome was divided into bins of a user-defined size (*B*), the frequency of reads mapped to each bin was determined and normalized for GC-content (using the LOWESS procedure). This GC-normalization improved aberration detection as it decreased the average allowed deviation of the read frequency per tested bin (Supplementary Table S4).

For every target bin, the squared Euclidean distance to every other bin was calculated by summing the squared GC-normalized read frequency differences over a set of normal samples (Figure 2b):
(1)
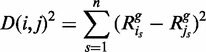

where 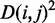 is the squared Euclidean distance between GC-normalized read frequencies of bins *i* and *j*, *s* defines the sample in the set of *n* reference samples and 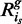 is the GC-normalized relative read frequency of bin *i* in sample *s*.

**Figure 3.**
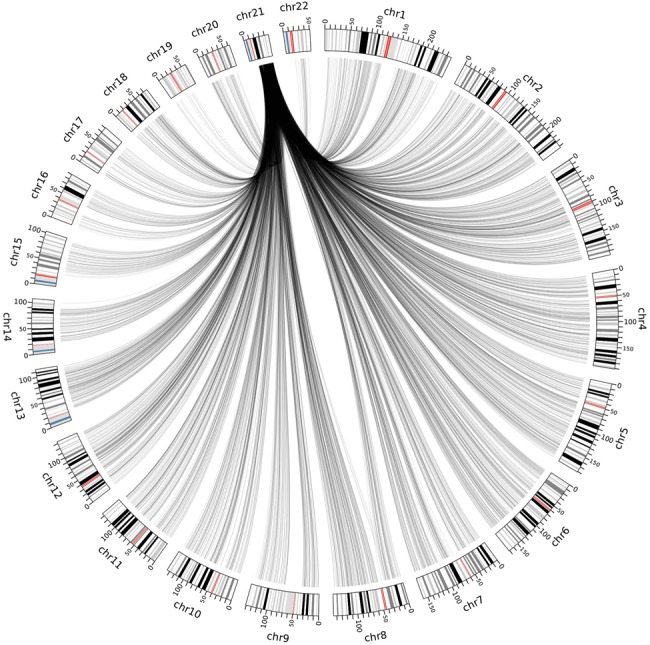
Overview of the selected reference bins for all target bins on chromosome 21. Cytobands are shown on the outside of the circle along with base pair positions in Mb and chromosome number. Lines connect target bins on chromosome 21 and their corresponding reference bins on other chromosomes. Opacity indicates the amount of overlapping lines. Barely any connections to chromosome 19 are made as the read frequency behavior of bins on chromosome 19 differs too much from the bins on chromosome 21 over different samples. Repeat rich regions such as the acrocentric p-arm of chromosome 21 and the regions surrounding the centromeres could not be mapped, and will therefore not be selected as reference bins. Image made using Circos (21).

For every target bin, the bins with the smallest distances were selected as reference bins. To avoid using a bin that is part of the same aberration as the target bin, no bins that are on the same chromosome as the target bin are selected as reference bins (Figure 2c). To circumvent obtaining a set of reference bins neighboring each other, which are likely to show the exact same behavior, all directly neighboring bins in a set of reference bins are removed except for the bin with the smallest distance. This initial set of reference bins is filtered by quality to ensure that no largely deviating bins are used for comparison. To do this, the best matching (smallest 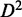) reference bin for every target bin is selected and the mean 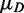 and standard deviation 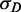 are determined over this set. Then, for every target bin, any reference bin with a distance to the target bin larger than 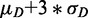 is removed from the reference set. This whole procedure is repeated once more for all target bins to improve quality, as the first time removes mostly far outliers (see supplement) that have a considerable impact on the estimates of 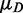 and 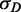. The pseudocode describing this filter is shown in Algorithm 1. As a result, most target bins are left with a set of good quality reference bins. Note that the amount of reference bins might be different for each target bin and some target bins may have no or few reference bins. Such target bins will be considered uncallable if the set of reference bins contains <10 bins. In the reference we built, 22.88% of the bins was considered uncallable. A plot showing the amount of reference bins per target bin after this step is depicted in Supplementary Figure S1, and an impression of the spread of reference bins for chromosome 21 is shown in Figure 3.


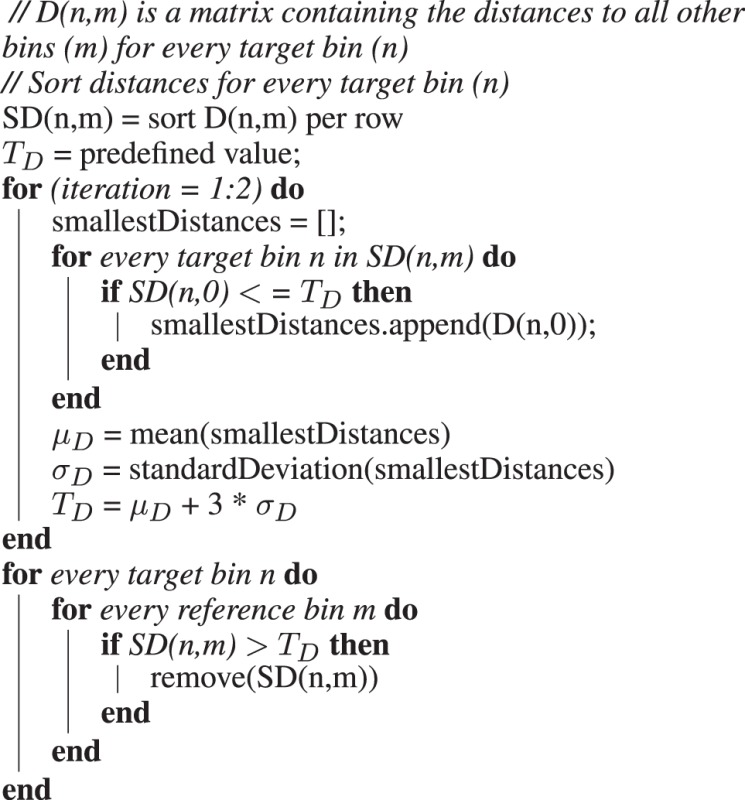


**Algorithm 1:** Pseudocode for the creation of a final matrix SD, containing only high-quality reference bins for all bins.

### WISECONDOR: subchromosomal scoring

For every sample, every bin was tested against its own set of reference bins within the same sample using a *z*-score method based on GC-normalized read frequencies. Classifications based directly on this method are further referred to as calls made by the ‘individual bin method’. Subchromosomal scores were eventually calculated by combining the *z*-scores of the individual bins within a sliding window using Stouffer’s *z*-score, denoted as the ‘sliding window method’.

#### Individual bin method

For every target bin in the test sample, the GC-normalized read frequencies of the set of previously defined reference bins within the same test sample are collected for the target bin of interest; the mean and standard deviation are calculated and used to determine the *z*-score:
(2)
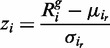

where *z_i_* is the deviation score for bin *i* in the test sample according to the individual bin method, 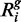 is the GC-normalized read frequency of the target bin *i* in the test sample, while 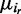 and 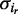 are the average and standard deviation of the GC-normalized read frequencies in the test sample of the bins in the reference set *i_r_* of the target bin *i*. If the absolute value of the *z*-score is ≥3, the bin is marked as potentially aberrated.

To improve sensitivity, we need to ignore aberrated bins in the reference set for each target bin. Therefore, the target bins found deviating (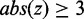) are stored in matrix *L*. Then, the *z*-score testing for every target bin is repeated without using reference bins that are in *L* [this affects the mean and variance in Equation (2)]. This step is repeated until *L* remains unchanged over two consecutive runs or a user-defined maximum amount of tests is done (to avoid oscillating behavior).

To remove excessive calls close to eachother, we allow small gaps within a detected region and still consider it one aberated region (*MaxBinSkip* in Supplementary Table S4). Also, to remove calls that result from few peaks that happen to be close to eachother, we put a threshold on the minimum amount of connected bins found aberrated before calling the aberration (*MinLength* in Supplementary Table S4). In the results presented here, we allowed gaps of at most 2 bins between any two such detected aberrated regions and required a minimum size of 10 bins to make a call.

#### Sliding window method

Since the individual bin method turned out to lack sensitivity (see ‘Results’ section) and suffered from peaks, we applied another approach based on a sliding window analysis. In this method, a bin is not tested in isolation, but instead, the *z*-scores of the bins neighboring the target bin are included in the aberration detection using Stouffer’s z-score:
(3)
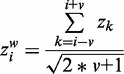

where 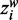 is the (sliding window) *z*-score for bin *i* using a window size that considers *v* bins on the left and right of bin *i*. A bin is now considered potentially aberrated when 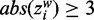, i.e.
(4)
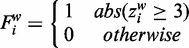

where 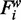 is the classification result for target bin *i* using the sliding window method. Spurious peaks and valleys are now ignored when the *z*-score is calculated for a region. When uncallable bins exist in the sliding window, their values are ignored, thus reducing the total amount of bins in the window. To reduce false positives caused by multiple peaks close to each other, we also applied the gap and minimum amount of bins filters as described in the individual bin method. In the results presented here we applied a sliding window of 11 bins (1 Mb each).

### WISECONDOR: chromosomal testing

Chromosomal aneuploidy detection is based directly on subchromosomal classification, as any aneuploid chromosome has nearly all of its bins marked as deviating by the sliding window method, while applying a chromosome-wide Stouffer’s *z*-score returned too many false positives (as shown in Supplementary Table S5). To detect whole-chromosome aberrations, we implemented a user-defined threshold *T* on the ratio of bins found deviating by the sliding window method:
(5)
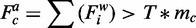

where 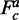 is the aneuploidy classification result for chromosome *c*, *m_c_* is the amount of callable bins on chromosome *c* and *T* is the user-defined threshold. In the presented results, we applied 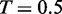 as threshold for chromosomal aneuploidy detection.

### WISECONDOR: bin sizes

Assuming a uniform and independent mapping of reads on the genome, the expected read depth 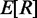 for bin *i* is as follows:
(6)
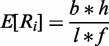

where *b* is the bin size used, *h* is the coverage of the sample, *l* is the read length and *f* the ratio of fetal DNA in the sample.

For a sample with an average coverage (*h*) of 0.5, while the read length is (*l*) 50 nt long, which is tested with bins that have a bin size (*b*) of 1 Mb, the expected amount of reads mapped to any bin is 10.000. Assuming a fraction of fetal DNA ( *f* ) of ∼5% (3), we expect 500 fetus-DNA–derived reads per bin. This seems a reasonable amount compared with expected natural fluctuations. Using bin sizes <1 Mb results in proportionally smaller numbers of reads per bin, complicating copy number detection in the data. Varying bin sizes indeed showed this and thus we selected bin sizes of 1 Mb. Larger bin sizes provided no significant improvements (data not shown), while losing detection resolution.

### WISECONDOR: sample quality assessment

Assuming that the percentage of fetus-derived DNA is ∼5% of the DNA in any maternal plasma sample (3), WISECONDOR should be able to detect read frequency differences of at least 5% to call a copy number change of a chromosomal region. To test whether WISECONDOR results are reliable enough, we determined the average allowed deviation over all bins (AvgASD in Supplementary Table S4). For every target bin, the standard deviation of the read frequencies within its reference bin set is divided by the read frequency of the target bin, resulting in a value that represents the minimum relative change in read frequency required to make a call for that target bin. When the mean of all these values (AvgASD) is >0.05, the results are considered less reliable since any aberration would require >5% read frequency difference to be detected. High AvgASD values seem to be independent of read coverage, but they are correlated with an increase of false-positive calls, especially on chromosome 19. GC-normalization resulted in a strong decrease in AvgASD values (Supplementary Table S4), thus improving detection ability. Although calls for samples with a high AvgASD are annotated as ‘unreliable’, the WISECONDOR script runs the standard tests and generally correctly identifies copy number aberrations.

## RESULTS

DNA was isolated from 56 blood plasma samples taken from pregnant women. All samples were assumed to contain at least 5% fetal DNA. This was checked for 14 maternal blood samples, all with male fetuses. The percentage of fetal DNA was measured using absolute quantitative polymerase chain reaction for *SRY* (fetal) and *HBB* (fetal and maternal), which showed the percentage of fetal DNA to be 7.6% (median). See supplement for more details and Supplementary Table S6 for the measured percentages in each sample. The isolated DNA was sequenced using the Solexa/Illumina HiSeq2000, split over three different runs (A, C and D), which were sequenced at different times. We obtained 51-bp single end reads with genomic coverages ranging from 0.04- to 1.66-fold, mostly between 0.2 and 0.7. The set of test samples contained eight different trisomy 21 (T21) cases, 2 trisomy 13 (T13) cases, 2 trisomy 18 (T18) cases and 2 trisomy 22 (T22) cases and four samples with subchromosomal deletions/duplications (Table 1). All samples were karyotyped to provide a ground truth. All samples were multiplexed for sequencing and demultiplexed with a maximum of one mismatch in their tags. The sequenced data were mapped to reference genome Hg19 GRCh37.65 using SOAP2 (22) with zero mismatches allowed, and all reads mapping to more than one genomic position were discarded.

**Table 1.** Overview of samples, read coverages and detected aberrations

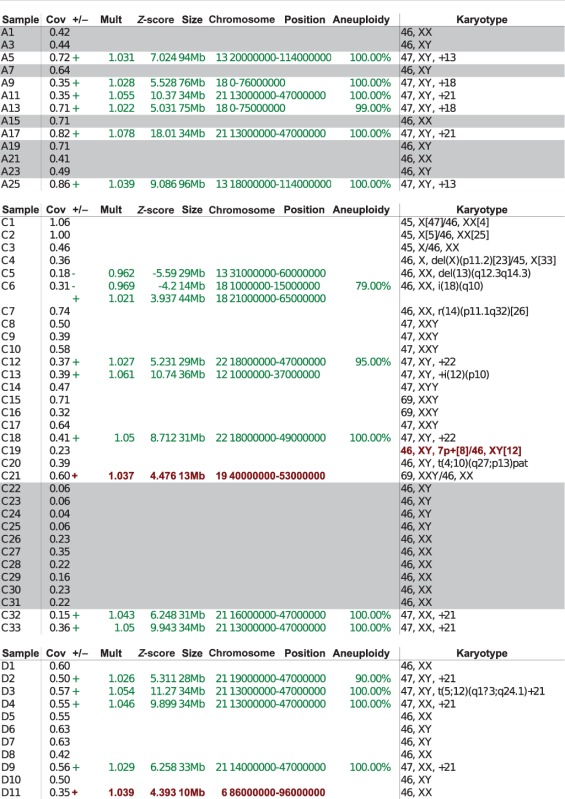

The samples with a gray background are the 17 samples used to build the set of reference bins. Samples with green text indicate samples in which the positions of a called copy number change correspond with the karyotyping analysis of CVS or AF (true positives). Samples with no position information denote samples for which no copy number change was detected by WISECONDOR or by karyotyping (true negatives). Samples with red text in the position columns indicate falsely called genomic regions (false positives), while red text in the karyotype column marks a missed aberrated region (false negative). A percentage in the aneuploidy column indicates the sample was called aneuploid, and the percentage shown is the amount of bins found deviating. The column denoted as Mult contains the average multiplication compared to the expected values for bins in the called region. No results <10 Mb are shown in this table. The rightmost column indicates the karyotyping based on CBS or AF.

### Data preparation

To remove extreme peaks with high read depths, all samples were filtered using a custom-designed filter called RETRO (Supplement: Retro Filter, Supplementary Figures S2 and S3). This filter removes all but the first read of strongly overlapping reads, as described in the supplement. Bin sizes of 1 Mb, 500 kb, 250 kb and 100 kb base pairs were tested. One mega base pair showed stable results, while smaller bin sizes suffered from noise and strong variations (data not shown). The GC-normalization was estimated per sample using Biopythons LOWESS function on the GC-count and read depth per bin (Supplementary Figure S4). In all steps, gender-specific chromosomes were omitted because the amounts of reads that map to the X and Y chromosomes are too strongly correlated with the percentage of fetal DNA in the sample as well as the fetus’ gender. If more reference samples would be available, detection of subchromosomal disorders on chromomes X could become possible. Detection of aberrations on the Y chromosome appears to be more problematic owing to its small size and repetitive sequence, leading to mappability difficulties.

### Aneuploidy classification

**Figure 4.**
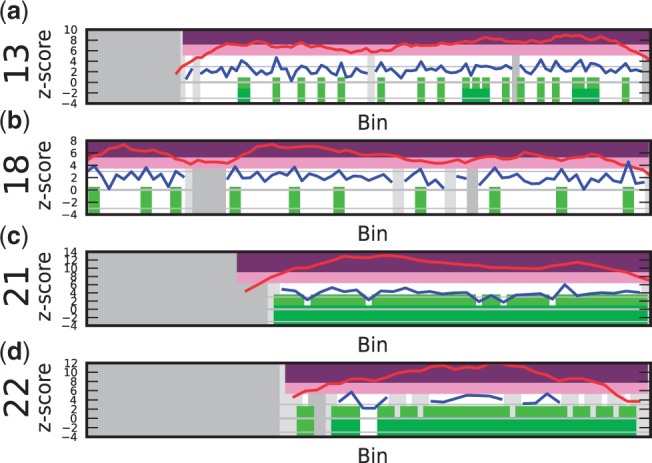
Samples with trisomies 13, 18, 21 and 22 demonstrate the difference in calling results from the sliding window method and the individual bin method. The vertical axis depicts the z-score, and the horizontal axis the bins on chromosomal positions. The blue line shows the z-score per bin, the red line plots the z-score using the sliding window (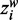). Purple regions show bins called by WISECONDOR (i.e. the sliding window approach). Dark green regions mark bins called with the individual bin method. Light green and pink regions are bins found deviating, by the individual bin and sliding window approach respectively, but those are too small in width such as spikes (and thus did not pass WISECONDOR’s minimum size requirement). Horizontal gray lines denote the *abs*(*z*) = 3 threshold. Gray regions are uncallable regions, where light gray is caused by being unable to find enough reference bins and dark gray regions are bins containing mostly unmappable (repetitive) sequences. (**a**) Sample A5: Trisomy 13. (**b**) Sample A9: Trisomy 18. (**c**) Sample A11: Trisomy 21. (**d**) Sample C18: Trisomy 22.

Using WISECONDOR, all samples with a trisomy for chromosome 13, 18, 21 or 22 were identified correctly with the sliding window method when we required that at least half of the bins on any chromosome were detected as a copy number change (Figure 4 and aneuploidy column in Table 1). Fetuses with a triploidy could not be classified correctly, as there are no genomic copy number changes relative to other chromosomes in the sample.

## Subchromosomal classification

**Figure 5.**
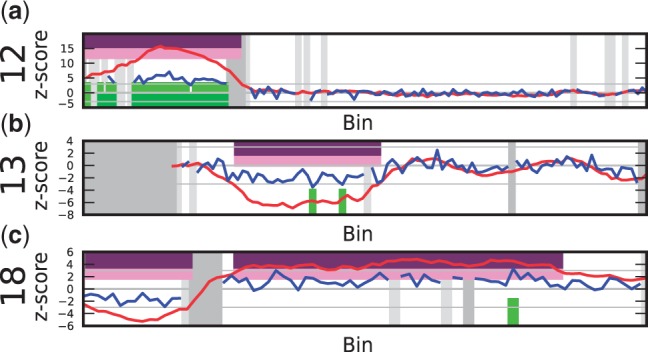
Output results for subchromosomal aberration detection (for an explanation of the plots, see Figure 4). (**a**) Sample C13: isochromosome 12p10. (**b**) Sample C5: deletion in the middle of the long arm of chromosome 13. (**c**) Sample C6: loss of the short arm on chromosome 18 (18p10) to the left and the gain of the long arm (18q10) to the right of the centromere. Note the difference in signal height between the two extra copies on chromosome 12p, and one extra copy on chromsome 18q.

Four (CVS) karyotyped samples of pregnancies of fetuses with autosomal subchromosomal disorders functioned as test cases for subchromosomal aberration detection:

*C13: 47,XY, + i(12)(p10).* DNA of this fetus had an extra isochromosome of the short arm of chromosome 12, resulting in four copies of 12p. Both the sliding window method and the individual bin method successfully identified the 12p copy number change from the centromere all the way to the end of the telomere (Figure 5a). The sliding window considers a group of bins instead of just the bin itself, resulting in a small overestimation of the aberrated region. This is to be expected when a diploid area juxtaposes an area with a copy number gain or loss.

*C5: 46,XX,del(13)(q12.3q14.3).* The fetus in this sample had a deletion of ∼30 Mb in the middle of the long arm on chromosome 13. Even though the sample had only 0.18-fold coverage, the deletion was called correctly by WISECONDOR using the sliding window method (Figure 5b). The individual bin method could not identify this deletion: it only called two deviating bins, which were ignored for being too far apart and too small in length (see ‘Materials and Methods’ section for details).

**Figure 6.**
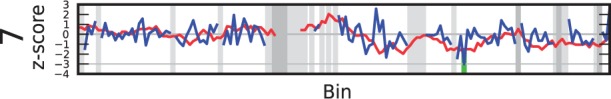
An example of a false negative (for an explanation of the plot see Figure 4). Sample C19: the 7p + [8]/[12] mosaic is not detected (also no considerable deviating *z*-scores of the bins, as indicated by the blue line, are noticable).

*C6: 46,XX,i(18)(q10).* This sample had an unbalanced translocation on chromosome 18, ie. the short arm of chromosome 18 was lost and replaced by an additional long arm. Both changes were correctly identified by WISECONDOR (Figure 5c). The individual bin method only found a single bin deviating, while WISECONDOR’s sliding window method marked almost the whole aberration. Note that, although still detectable, these single-copy changes resulted in much less deviating *z*-scores than the two additional short arms in the C13 case.

*C19: 46,XY,7p + [8]/46,XY[12].* The DNA of the fetus in this sample had a mosaic containing an additional short arm of chromosome 7 in 40% of the karyotyped cells. This chromosome arm gain could not be identified with WISECONDOR (Figure 6). The combination of this mosaic with the fetal DNA percentage and the low coverage of the sample may have caused the aberration to be too subtle in read frequency to be identified correctly using this method.

*Other samples.* As expected, balanced genetic changes such as the translocations in samples C7, C20 and D3 were not found using this method.

**Figure 7.**
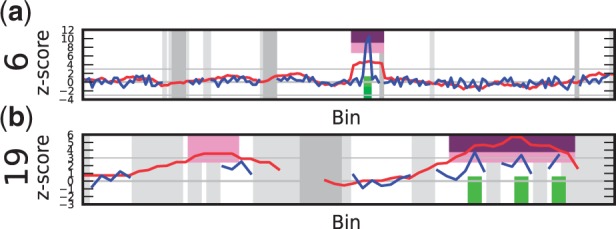
Examples of false positives (for an explanation of the plot see Figure 4). (**a**) Sample D11: false positive on chromosome 6. A 2 Mb sized peak is spread out due to the sliding window method. Additional testing proved this was caused by a maternal CNV. (**b**) Sample C21: false positive on chromosome 19. Note the lack of data points (testable bins) as can be seen from the shape of the blue line and the large amount of gray areas, which is most likely caused by the different GC-content in chromosome 19 compared with other chromosomes.

*False positives.* Although most of the calls made by WISECONDOR comply with the known disorders for the tested samples, it also generates some false positives (Table 1). The false positives are relatively small regions, with the largest one being 13 Mb. Increasing the minimum size of aberrations directly decreases the number of false positives: using a minimum size of 20 Mb, WISECONDOR rendered no false positives but failed to detect one known aberration. One false positive appeared in Sample D11 on chromosome 6 (Figure 7a). The peak itself is two bins wide, and therefore it was not removed by WISECONDOR and instead reported wider than it actually was. Running WISECONDOR on the same sample sequenced by another facility resulted in nearly the same peak, leading us to believe that the peak actually exists in the sample and, considering the height, it is most likely maternal. Further testing pointed out there was a maternal gain of 636 kb, which covered the area we expected based on the size of the peaks. Therefore, this false positive is due to a maternal aberration and was not expected to be filtered out.

Chromosome 19 proved to be a difficult chromosome for WISECONDOR. With increasing sensitivity, numerous false positives occur on chromosome 19. This is most likely caused by its GC-richness, making it difficult to find bins that behave alike on other chromosomes, even after GC-correction. One example of a false positive on chromosome 19 in our test data is shown in Figure 7b). The reported areas on this chromosome are no known CNV regions according to the Database of Genomic Variants although they do overlap with several smaller known CNV’s.

## DISCUSSION

We have demonstrated that both chromosomal and subchromosomal aberrations can be determined by within-sample comparison of bin read frequencies instead of using a set of re-sequenced reference samples.

We showed that the developed method, WISECONDOR, is sensitive enough to detect the small alterations in read frequency caused by copy number aberrations in the fetal DNA, using shallow sequencing. These relative changes are assumed to be >5%, equal to the expected percentage of fetal DNA in maternal plasma samples (which was confirmed for 14 samples, Supplementary Table S6). The results show that any aberration >13 Mb that we tested was correctly called, whereas false positives were never >13 Mb. Even for the relatively small amount of reads we used, this method provides nearly the same precision as karyotyping would, for which detection of small aberrations is limited to ∼10 Mb owing to the resolution of imaging. The main reason for the limited resolution of WISECONDOR (and this kind of tests in general) is the natural variations in read depth per genomic region. This may be caused by biological differences such as GC-content or repetitive sequences in the DNA, making the detection limit of duplications and deletions dependent on their genomic locations. The influence of these biological differences becomes more pronounced with decreasing sequencing depth.

As expected, chromosome 19 was more prone to false-positive results than other chromosomes, as it is known to have a different GC-content compared with other chromosomes. It is noteworthy that a putative small false positive we detected on chromosome 6 turned out to be of maternal origin. This means that even with the limited amount of reads we have used, it is possible to obtain unsollicited findings in the form of maternal CNVs. This might have consequences for counseling of pregnant women undergoing Non-Invasive Prenatal Testing (NIPT). WISECONDOR is based on the assumption that most of the genome is unaberrated. Therefore, it is not possible to do within-sample comparison if most of the genome is aberrated, as in triploidy cases. Additionally, some samples have strongly fluctuating read frequencies, making within-sample comparison unreliable. To identify such samples, WISECONDOR provides a warning when it is unlikely to detect read frequency changes as small as 5%.

As sequencing becomes more affordable, higher quality data will become available quickly without increasing sequencing cost. This allows for more precise diagnostics as the method developed here is expected to perform better with increased sequencing depth of both reference and test samples. A higher coverage will allow for more stable calls as well as using smaller bin sizes while keeping the read depth per bin high enough to detect changes confidently.

Taken together, WISECONDOR is able to detect subchromosomal and chromososmal disorders with the exception of triploid and mosaic cases at low sequencing coverage per sample. It thereby allows noninvasive prenatal diagnotiscs without increasing costs compared with current practices for trisomy 21 detection. Although additional studies are necessary to validate WISECONDOR in a true diagnostic setting, WISECONDOR may prove a valuable asset in prenatal diagnostics as an objective noninvasive routine test for pregnancies. The data used showed promising results for coverages as low as 0.18-fold, although 0.3-fold coverage is a more reliable baseline in our experience. At the time of writing, this coverage can be achieved by multiplexing eight samples on a single lane using the HiSeq2000, bringing costs as low as $300 per sample.

## SUPPLEMENTARY DATA

Supplementary Data are available at NAR Online.

## FUNDING

Funding for open access charge: VU University Medical Center, Department of Clinical Genetics.

*Conflict of interest statement*. None declared.

## Supplementary Material

Supplementary Data
