# Supplementary material for: WISECONDOR: detection of fetal aberrations from shallow sequencing maternal plasma based on a within-sample comparison scheme
Source: Nucleic Acids Res. 2013 Oct 28;42(5):e31. doi: 10.1093/nar/gkt992 (PMC3950725; doi:10.1093/nar/gkt992)
Supplement: Supplementary Data [file supp_42_5_e31__index.html]

WISECONDOR: detection of fetal aberrations from shallow sequencing maternal plasma based on a within-sample comparison scheme — WISECONDOR: detection of fetal aberrations from shallow sequencing maternal plasma based on a within-sample comparison scheme — Supplementary Data 

# WISECONDOR: detection of fetal aberrations from shallow sequencing maternal plasma based on a within-sample comparison scheme

## Supplementary Data

files

**Files in this Data Supplement:**

- Supplementary Data - pdf file
