# Supplementary material for: WISECONDOR: detection of fetal aberrations from shallow sequencing maternal plasma based on a within-sample comparison scheme
Source: Nucleic Acids Res. 2013 Oct 28;42(5):e31. doi: 10.1093/nar/gkt992 (PMC3950725; doi:10.1093/nar/gkt992)
Supplement: Supplementary Data [file supp_gkt992_nar-01466-met-n-2013-File016.pdf]

SUPPLEMENT
WISECONDOR: Detection of fetal aberrations from shallow sequencing maternal blood based on a within-sample normalization scheme.

R. Straver [1,2] *, E.A. Sistermans [2], H. Holstege [2], A. Visser [3], C.B.M. Oudejans [3], and M.J.T. Reinders [1,2]

[1]Delft Bioinformatics Lab, Delft University of Technology, Mekelweg 4, 2628 CD Delft, The Netherlands.
Departments of [2]Clinical Genetics and [3]Clinical Chemistry, VU University Medical Center Amsterdam, van der Boechorststraat 7 (BS7/J377), 1081 BT Amsterdam.

RETRO FILTER

Plotting read depth per bin revealed extremely large peaks in the data, sometimes referred to as read towers. These towers varied greatly in size among samples and proved troublesome for normalization of the data as their height influenced the normalized values of other regions greatly. They appeared on some but not all chromosomes. The largest peaks were generally found near centromeres. But other smaller peaks also appeared in other areas although only in some samples. Removing these areas did not seem sufficient as the positions of the smaller peaks varied too much among samples. Close inspection of the mapped reads in the read tower regions showed many duplicate reads. Removing exact duplicates did not prove sufficient as the read towers were still partly there. Further analysis of the towers showed that the reads that were left after filtering were all strongly overlapping. As the coverages of samples used for this study were very low (often about 0.3 fold), the RETRO (REad Tower RemOval) filter was created using this knowledge.

For every read in the data, if the next read starts within W base pairs after the current read, this read is added to the stack of reads that make up the current tower. Then, the newly added read is used to determine the distance to the next read and the previous step is repeated. If the next read does not start within W base pairs, the stack is flushed and the containing reads are either kept whenever the tower was less than a predefined threshold T, or removed except for the first read in the stack if it contains more than T reads.

To find an appropriate setting for the read tower threshold, we described the probability for any tower to happen in a random dataset. First, to determine the probability for reads to be within range W from eachother, we assume the reads to be spread uniformly across the genome. Then, the number of reads r within a certain window W can be modelled by a Poisson distribution:

$$P_{R(W)}(r) = \frac{(\lambda W)^r e^{-\lambda W}}{r!} \quad (1)$$

with

$$\lambda = \frac{t}{G} \quad (2)$$

where λ describes the mean distance between any two adjacent read start positions, t describes the total amount of reads and G the genome length.

The probability of observing any read within a window of N is equal to 1 minus the chance of observing no reads within that window, thus:

$$1 - P_{R(W)}(0) = 1 - \frac{(\lambda W)^0 e^{-\lambda W}}{0!} = 1 - e^{-\lambda W} \quad (3)$$

We consider a series of reads a read tower when this series has at least T sequential reads within range W of eachother. The chance for any read to start such a tower is equal to $1 - P_{R(T)}(0)$ happening T times in a row:

$$P_{tower} = (1 - e^{-\lambda W})^{T-1} \quad (4)$$

where T defines the threshold to consider a stack of reads a tower that should be removed. We introduced -1 because we always start from a read. If we combine everything into a single equation we end up with:

$$P_{tower} = (1 - e^{-\frac{t}{G} W})^{T-1} \quad (5)$$

Using this formula we can approximate the expected amount of times we remove a set of reads that existed at random instead of being part of an actual read tower for any setting of W and T.

Settings used for this work were a maximum distance of $W = 4$ between read start positions and a threshold at $T = 4$ reads.

*To whom correspondence should be addressed. Tel: +31 20 44 48346; Fax: +31 20 44 48293; Email: r.straver@vumc.nl

QUALITY FILTER THRESHOLD

The quality threshold T_D is repeated several times to the improve quality of used reference bins, removing the strongest ouliers in the first cycle and relatively small outliers in the cycles after that. For the reference set used, the first 10 cycles resulted in the following threshold levels. Note the quickly decreasing difference between succeeding cycles at the start. We only applied 2 steps instead of the 10 shown here for illustration.

0. 0.0284748882881
1. 0.0101273306611
2. 0.00825977598937
3. 0.00773579488437
4. 0.00749311500216
5. 0.00736220232047
6. 0.00728857040126
7. 0.00727280004711
8. 0.00727280004711
9. 0.00727280004711
10. 0.00727280004711

SAMPLE COLLECTION

Pregnant females (n= 3500) were recruited between 2003-2012 as part of non-invasive prenatal research studies at the VU University Medical Center, Amsterdam, approved by local research ethics committee. Written informed consent was obtained from the patients. Blood samples were obtained in EDTA tubes (3x7 ml) during first trimester (week 9-14) prior to invasive procedures (chorionic villus sampling or amniocentesis) and centrifuged within 6 hours for 3,000 rpm at 4oC to separate the plasma from the peripheral blood cells. The plasma was then further centrifuged at 12,000g for 10 min at 4°C to pellet any remaining cell fragments. The purified plasma was stored as 800 ml aliquots at -80°C.

AUTOMATED DNA ISOLATION

Plasma DNA (4 ml per patient) was isolated on the BioRobot MDx (Qiagen) with a customized protocol. Input was increased 3-fold, no carrier RNA was added and elution was done in MilliQ water. DNA isolates from the same patient were pooled, and concentrated by SpeedVac.

FETAL DNA FRACTION

The fetal DNA fraction was calculated by absolute quantitative PCR for SRY (fetal) and HBB (hemoglobin B) (fetal and maternal) using 5 microliters of the same cDNA libraries as used for MPS. The absolute quantities were converted to genomic equivalents (GE) using a conversion factor of 6.6 ng/cell. To correct for the haploid/diploid difference, the SRY GE was corrected by a factor 2. Although the HBB measurement contains fetal message as well, this was left uncorrected. The Fetal DNA percentage was calculated by using the formula (2 x SRY GE/HBB GE) x 100.

Sample	SRY ng	HBB ng	Fetal Fraction (%)
**A5**	0.441	20.712	4.258
**A6**	0.128	8.506	3.010
**A8**	0.122	7.063	3.455
**A10**	0.116	4.466	5.195
**A12**	0.155	6.204	4.997
**A13**	0.528	14.037	7.523
**A14**	0.996	23.413	8.508
**A17**	0.673	14.240	9.452
**A18**	0.498	7.410	13.441
**A19**	1.135	15.905	14.272
**A20**	0.367	11.596	6.330
**A24**	0.782	20.431	7.655
**A25**	0.916	14.649	12.506
**A26**	0.629	12.274	10.249
**Median**			7.589

Table S6. Overview of measured fetal DNA fractions. The even numbered samples shown correspond to the samples that are part of set A in the paper.

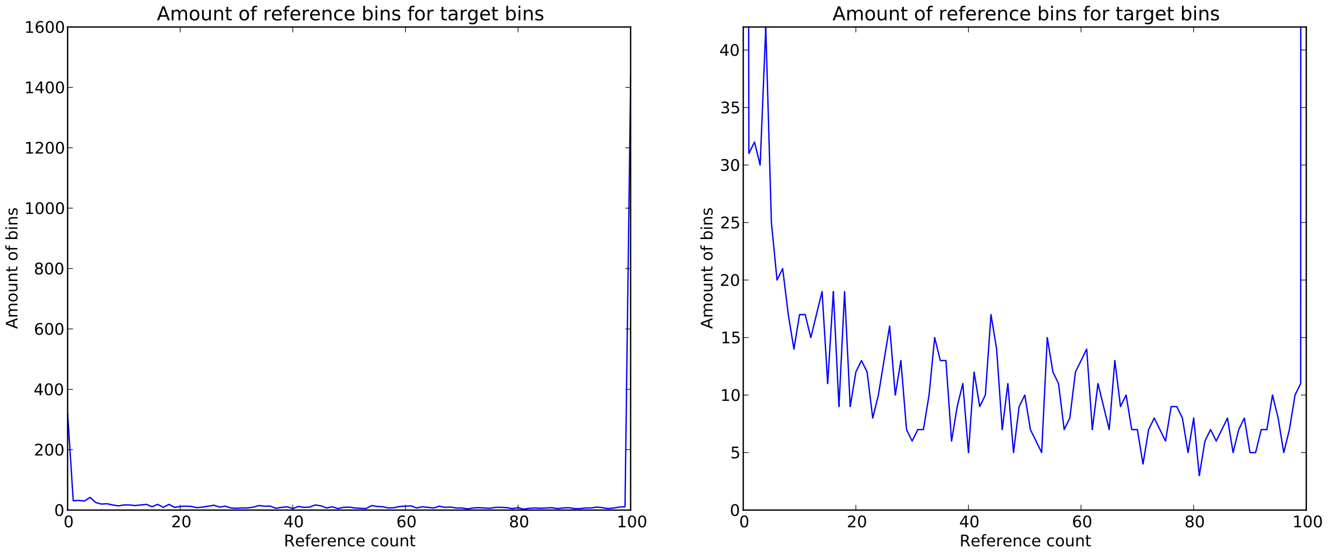

Figure S1. Plots of the amount of reference bins per target bin after the reference bin quality filter. The left figure shows the original data, the right figure shows the same plot without the values 0 and 100 to show the distribution inbetween in more detail. The large spike at 100 means there are at least 100 reference bins available for the target bins. As can be seen here, most target bins have either 100 reference bins, the maximum, or no reference bins at all.

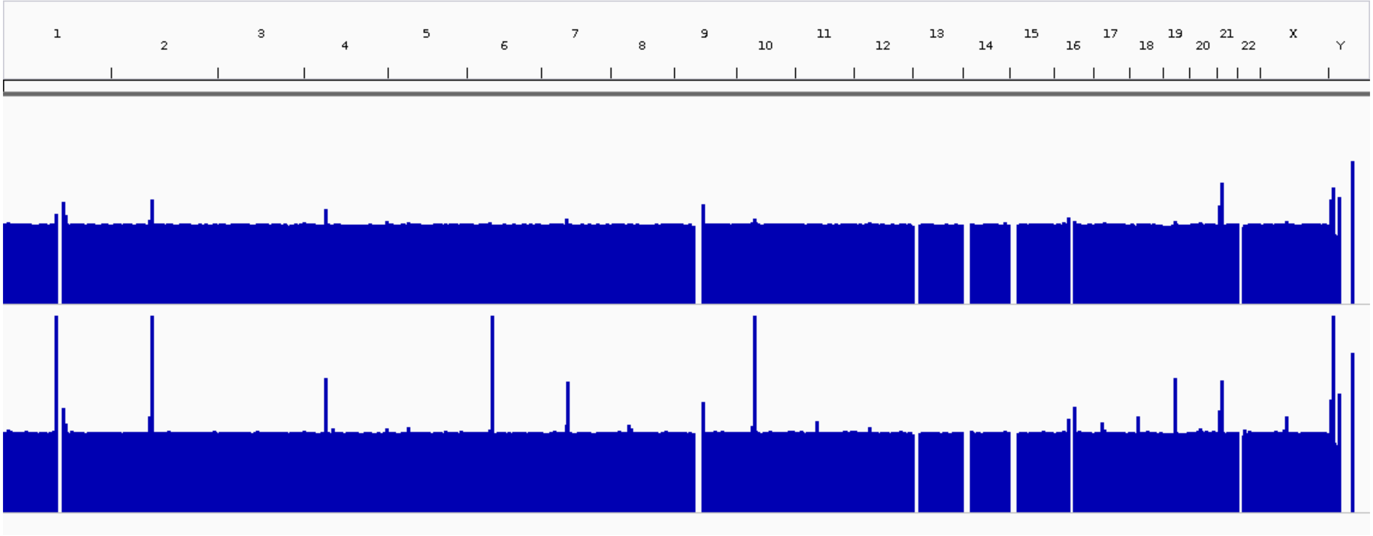

Figure S2. Overview of all read depths of C28 using IGV. The x-axis shows the position on the genome while the y-axis shows the read depth. Chromomes are specified at the top, the resulting data after RETRO filtering is shown in the middle. The original data before filtering is shown at the bottom. As is visible, most of the read towers are removed almost completely.

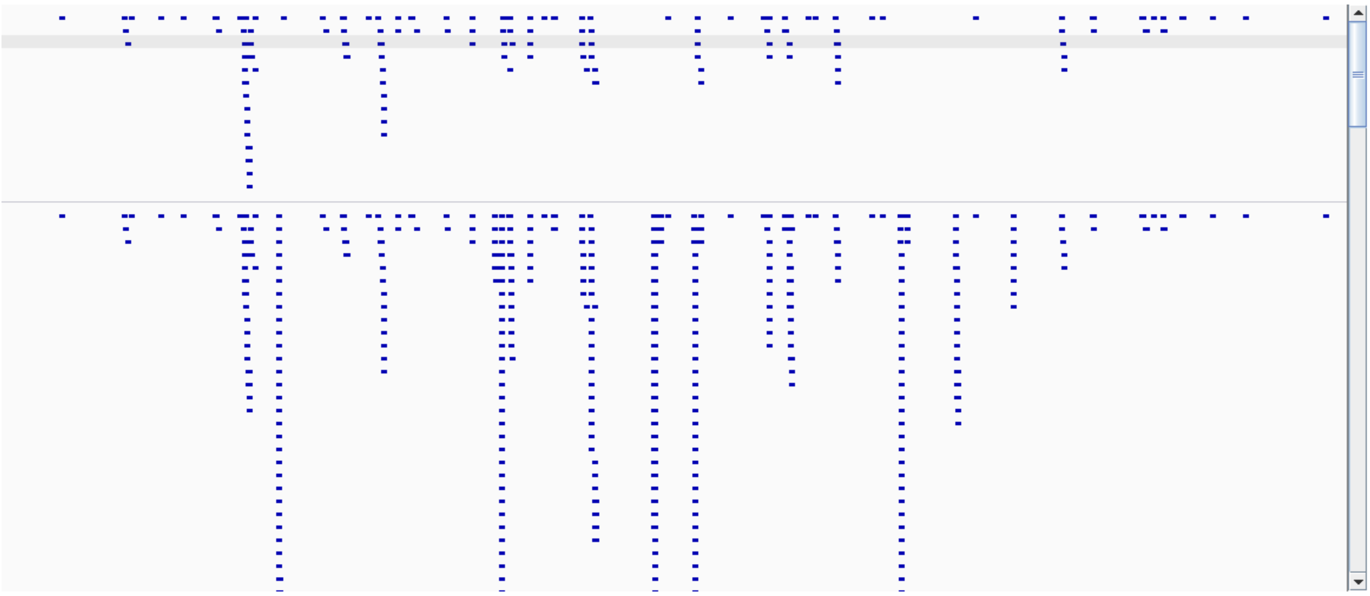

Figure S3. Close up of a read tower area using IGV. The area shown here is part of the read tower on chromosome 1, C28. The top figure shows the result after RETRO filtering. The bottom figure is clipped. As shown, numerous reads that seem to have a normal spread are kept while the extremely large stacks between them are removed.

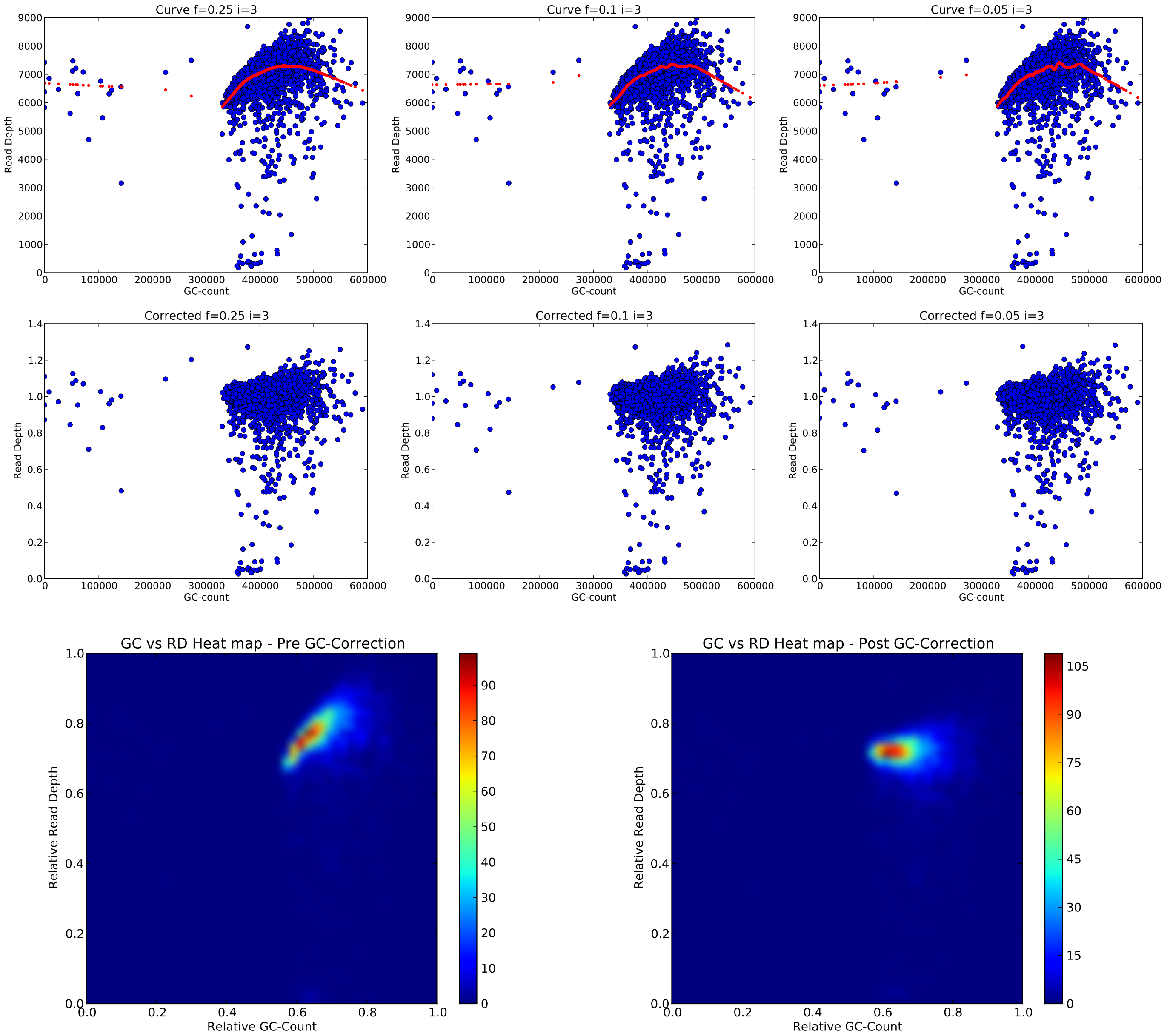

Figure S4. Plots of several LOWESS settings for GC-correction on sample C16. The figures in the top show the original data in blue with the LOWESS fitted points in red. The figures in the middle row show the corresponding results after using the LOWESS fit to correct the data. GC-count per bin is shown on the x-axes while the read depth is shown on the y-axis. Note the changed shape of the cloud of data points after correction compared to the original data. The figures in the bottom row show heat maps of the same sample, the left shows pre-GC-correction while the right plot shows the result of the LOWESS correction using f=0.1 and i=3.

	1	2	3	4	5	6	7	8	9	10	11	12	13	14	15	16	17	18	19	20	21	22	Karyotype
**A1**	3.25	2.82	2.33	-0.44	-2.42	2.94	-2.32	-2.30	-1.82	2.65	-1.72	0.35	0.38	-0.25	1.68	-2.94	2.19	-1.19	-3.19	-3.17	2.15	1.68	46,XX
**A3**	1.04	1.58	0.12	0.70	-1.19	1.59	-0.90	-1.43	-0.09	1.49	-2.14	0.86	0.98	-0.56	0.98	-1.84	1.07	-0.92	-1.45	-0.58	1.91	1.17	46,XY
**A5**	-0.63	-0.70	-0.69	-1.01	-0.11	-0.75	-1.01	-0.12	0.13	-1.21	-0.06	-1.00	9.47	-0.09	0.88	0.15	-1.00	-0.43	0.00	0.78	0.25	0.08	47,XY,+13
**A7**	-0.42	-0.24	0.94	-1.52	0.87	-0.14	-0.13	0.78	0.18	0.09	0.95	-0.83	-0.26	0.27	-0.25	0.30	-1.61	0.97	0.06	0.77	0.01	-0.08	46,XY
**A9**	-0.42	-0.47	-1.03	-1.38	-0.34	-0.52	0.28	-1.12	-1.37	-0.15	-0.51	-0.63	0.25	-0.08	-0.14	0.11	-0.35	8.35	0.42	0.69	-0.01	0.08	47,XY,+18
**A11**	0.19	0.28	-1.16	-1.08	0.76	-0.05	-0.33	-1.04	-1.18	-0.70	1.40	-1.01	-0.52	-0.54	0.61	-0.31	-0.14	-0.84	-0.54	0.18	11.94	0.21	47,XY,+21
**A13**	0.31	-0.05	0.63	-0.36	-0.57	0.06	-1.55	-1.40	-0.39	-0.29	-0.31	-0.72	0.27	-0.02	1.60	-0.51	-0.71	6.34	-0.42	-0.50	0.17	0.61	47,XY,+18
**A15**	-0.94	-1.30	0.91	0.46	1.11	-0.60	0.71	0.36	-0.18	-1.60	0.40	0.00	0.58	-0.28	0.25	0.47	-1.01	0.55	0.93	0.55	0.76	-0.66	46,XX
**A17**	-0.84	-0.68	0.36	-0.29	0.63	-0.71	-0.67	-0.41	0.60	-0.50	-0.21	-0.60	0.06	-0.27	0.20	-0.34	-1.33	0.27	0.20	-0.20	16.25	-0.84	47,XY,+21
**A19**	-0.35	-0.32	0.77	0.55	0.66	0.31	0.50	-0.54	-0.02	-0.72	0.60	-0.67	1.34	0.12	-0.59	-0.19	-1.18	-0.02	0.37	-0.24	-0.37	-0.25	46,XY
**A21**	-0.27	-0.47	-0.23	-0.97	0.53	-0.81	-0.17	-1.18	0.59	-0.93	0.53	-0.73	0.03	-0.31	1.82	0.31	-0.32	0.99	0.26	1.22	0.32	1.45	46,XX
**A23**	-0.30	-0.05	0.91	1.05	0.97	0.22	0.85	0.41	0.62	-0.32	-0.41	1.76	-0.77	-0.11	-2.04	0.33	-1.74	-0.54	0.02	-0.63	-0.04	-0.67	46,XY
**A25**	-0.80	-0.92	-0.49	-0.56	0.12	-0.57	-0.28	-0.80	-0.30	-1.49	-0.41	-0.27	12.28	-0.25	0.09	-0.34	-0.67	-0.30	0.31	-0.96	-0.34	0.12	47,XY,+13

	1	2	3	4	5	6	7	8	9	10	11	12	13	14	15	16	17	18	19	20	21	22	Karyotype
**C1**	-0.11	-0.76	-0.50	0.19	-0.22	-0.76	0.04	-0.54	0.44	-0.71	0.21	-0.02	0.03	0.19	-0.07	0.34	0.66	0.71	0.47	1.02	-0.70	0.36	45,X[47]/46,XX[4]
**C2**	-0.30	-0.81	-0.58	-0.50	0.78	-0.81	-0.02	-0.17	0.26	-0.73	-0.23	0.58	-0.16	-0.20	0.25	0.47	0.13	-0.40	1.16	0.75	-0.52	-0.22	45,X[5]/46,XX[25]
**C3**	-0.41	-0.60	0.70	0.26	-0.33	-0.69	0.53	0.44	0.26	-0.52	-0.21	-0.38	0.11	0.20	0.42	0.58	-0.84	0.61	0.23	0.60	-0.33	-0.02	45,X/46,XX
**C4**	-0.59	0.15	-1.13	-1.76	0.33	-0.28	0.28	1.26	0.01	-0.96	1.16	0.73	-0.08	0.90	-0.39	0.68	-0.95	-0.54	0.35	0.15	0.07	-0.42	46,X,del(X)(p11.2)[23]/45,X[33]
**C5**	0.33	-0.58	-0.59	-0.77	-0.19	0.49	0.04	0.16	0.26	1.21	0.50	-0.08	-4.83	0.22	-0.16	1.11	0.27	-0.15	0.06	0.79	-1.04	0.10	46,XX,del(13)(q12.3q14.3)
**C6**	0.10	-0.36	0.08	-0.11	-1.19	0.39	-0.96	-0.76	-0.55	-1.19	-0.97	0.93	0.21	-0.57	0.37	0.22	1.06	2.48	0.40	0.09	-0.39	0.66	46,XX,i(18)(q10)
**C7**	-0.37	0.05	-0.43	0.39	-0.08	-0.75	0.34	0.15	0.08	0.45	0.55	-0.60	0.06	-0.47	0.18	0.06	0.60	-0.21	0.17	0.22	0.09	0.71	46,XX,r(14)(p11.1q32)[26]
**C8**	-1.22	-0.23	0.05	4.19	1.61	1.46	1.60	3.03	-1.05	-0.42	-1.60	2.54	-1.88	0.01	-1.01	-0.04	-2.88	0.85	-0.56	-2.24	-1.83	1.35	47,XXY
**C9**	0.15	0.44	0.73	0.12	0.27	-0.05	0.01	-0.90	-1.20	0.36	-0.49	-0.24	0.28	0.13	0.06	-0.31	0.00	-0.42	0.06	0.07	-0.47	0.44	47,XXY
**C10**	0.02	0.06	1.09	1.01	0.29	-0.73	0.75	0.46	-0.49	-1.00	-0.95	-0.26	0.33	0.11	-0.87	-0.21	-0.05	-0.49	0.42	0.00	-0.46	0.48	47,XXY
**C12**	-0.70	-0.76	-0.64	-0.54	0.71	-0.12	-0.06	-0.08	-0.09	0.07	-0.49	0.31	0.55	0.11	-1.00	0.18	-0.01	-0.65	0.58	-0.23	0.87	4.98	47,XY,+22
**C13**	-0.01	-0.43	-1.21	-1.24	-0.67	-0.31	-1.05	-0.40	-0.76	0.21	0.26	6.61	-0.11	-0.56	1.05	0.30	-0.62	0.01	-0.19	0.41	-0.11	0.30	47,XY,+i(12)(p10)
**C14**	-0.75	-0.99	0.73	1.25	0.40	-0.41	-0.01	0.85	-0.09	-1.04	-0.06	-0.07	0.83	0.68	-0.22	0.21	-0.73	1.31	0.38	0.12	-0.21	-0.59	47,XYY
**C15**	0.12	0.05	1.35	-2.37	0.57	-0.60	-0.54	-0.05	0.04	-0.60	1.04	0.24	-1.03	-0.17	0.78	0.21	-0.40	-0.29	0.23	0.95	-0.03	0.29	69,XXY
**C16**	-0.28	0.15	1.21	0.16	0.48	-0.31	0.32	0.21	1.85	-0.41	-0.67	-0.22	-0.25	-0.26	-0.29	0.03	-0.21	-0.85	0.21	0.14	0.15	-0.72	69,XXY
**C17**	-0.71	-0.82	0.43	-1.22	0.88	-0.09	1.54	0.71	-0.49	-1.34	0.50	0.03	-0.50	-0.51	-1.02	0.86	0.38	-0.64	1.48	0.58	-0.95	-2.02	47,XXY
**C18**	-0.77	-0.60	0.42	0.10	-0.61	-0.34	1.08	0.07	-0.83	0.29	-1.07	-0.64	-0.19	-0.51	-0.70	0.01	-0.76	0.15	0.50	0.24	0.32	8.43	47,XY,+22
**C19**	-0.57	-0.66	-1.41	0.43	0.75	-0.73	0.27	-0.17	-0.61	-0.66	0.04	0.84	1.14	0.19	-0.18	-0.09	0.86	-0.16	0.86	0.42	0.50	-0.37	46,XY,7p+[8]/46,XY[12]
**C20**	-0.55	-0.55	-2.14	-0.77	-0.19	-0.66	-0.35	0.21	1.16	0.71	-0.14	-0.27	-0.40	-0.31	0.25	0.73	1.33	0.09	0.55	1.38	0.42	0.39	46,XY,t(4;10)(q27;p13)pat
**C21**	0.02	-1.21	-1.12	-2.40	1.15	-1.07	-0.65	0.61	-0.14	-1.36	1.76	0.89	-0.71	-0.89	-1.15	0.90	0.84	-0.92	1.78	1.85	-1.62	0.24	69,XXY/46,XX
**C22**	0.24	-0.26	-0.52	0.34	0.01	0.77	0.52	1.12	0.36	0.11	-0.21	-2.20	2.35	-1.20	0.54	-0.18	0.03	0.77	-0.49	-0.55	0.26	-1.21	46,XY
**C23**	-0.25	-0.23	-0.90	1.69	0.23	-0.30	0.21	1.82	-0.55	0.54	-0.65	-0.74	-1.02	0.01	-0.01	0.18	0.11	-1.25	0.72	0.61	-1.42	-1.45	46,XY
**C24**	-0.74	-0.35	-2.45	1.97	-0.81	-0.74	1.34	-0.59	2.31	-0.70	2.32	0.36	-1.00	1.80	-1.11	1.06	0.24	-1.69	0.39	-0.67	-1.79	-1.41	46,XY
**C25**	1.26	1.51	-0.16	-0.25	-1.34	-0.39	-2.41	0.07	1.32	1.21	0.06	0.57	-2.24	-0.71	-0.34	-0.48	0.29	0.53	-0.78	1.16	-1.19	1.51	46,XY
**C26**	-0.47	-0.36	-0.07	-0.25	1.08	0.27	0.63	-0.13	0.20	-0.88	-0.02	0.69	-0.36	-0.14	-1.70	0.84	-0.58	-0.54	0.77	-0.19	0.09	-1.12	46,XX
**C27**	-0.21	-0.17	-0.34	0.25	-0.58	-0.08	-0.04	0.16	-0.56	-0.19	-0.44	0.62	0.14	-0.43	0.47	0.21	0.19	0.21	0.39	0.14	-0.34	0.36	46,XX
**C28**	0.02	0.13	0.16	-1.75	-0.37	-0.54	0.40	0.08	1.06	-0.10	0.71	1.15	0.06	-0.45	-0.18	-0.37	1.27	0.00	0.30	-0.09	-0.51	-0.22	46,XX
**C29**	-0.16	-0.65	-0.91	-0.96	0.74	-1.31	0.13	0.29	-1.15	-0.50	-0.72	0.99	-0.01	-0.63	0.66	1.23	0.54	-1.12	1.00	0.21	-0.06	0.31	46,XX
**C30**	-0.90	-0.85	-0.54	-0.60	-0.55	-1.07	-0.21	-0.30	-1.11	0.34	0.06	-1.15	-0.51	3.20	-0.26	0.45	0.68	1.85	0.53	1.03	0.74	0.63	46,XX
**C31**	-0.81	-0.81	-0.03	-0.27	1.05	-0.11	0.88	1.40	-1.15	-0.51	0.68	-1.03	0.32	-0.35	0.08	0.62	-0.15	1.39	0.16	0.42	-0.51	-0.03	46,XX
**C32**	-0.35	0.69	-1.13	-1.74	-0.11	-0.03	-0.88	-0.46	-1.21	-0.65	0.65	1.43	-1.38	-0.10	0.96	-0.07	-0.15	-0.55	-0.20	0.70	8.58	0.85	47,XX,+21
**C33**	-0.58	-0.11	-1.71	-1.17	-0.18	0.08	-0.36	0.14	-0.85	0.11	-0.52	-0.06	-0.31	-0.53	0.03	0.41	0.15	-0.36	0.48	-0.30	10.76	-0.71	47,XX,+21

	1	2	3	4	5	6	7	8	9	10	11	12	13	14	15	16	17	18	19	20	21	22	Karyotype
**D1**	0.36	0.34	2.06	-0.16	-0.06	0.33	-1.41	-0.49	-0.46	0.26	1.51	-0.16	0.36	-0.20	0.30	-0.49	-0.73	-0.18	-0.54	0.18	-0.44	0.10	46,XX
**D2**	-0.05	-0.20	-0.47	-1.76	0.64	-0.48	-0.85	-1.71	-0.17	0.12	1.12	0.50	-0.28	-0.98	-0.78	0.64	0.51	-1.46	0.61	1.00	4.80	0.29	47,XY,+21
**D3**	0.53	0.38	2.08	-1.03	-0.74	0.38	-1.70	-0.19	-0.66	-0.23	-0.14	-0.50	-1.07	-0.63	0.73	-0.21	0.03	-1.41	-0.72	-0.65	11.74	-0.10	47,XY,t(5;12)(q1?3;q24.1)+21
**D4**	-0.26	-0.27	-0.96	-1.40	0.02	-0.83	-0.50	-0.67	0.26	-0.28	0.01	0.16	-1.02	-0.10	-0.52	0.18	0.10	-0.39	0.29	1.11	9.80	1.05	47,XX,+21
**D5**	-0.37	-0.79	1.53	-0.15	0.33	-0.54	-0.55	0.31	-0.03	-0.77	-0.73	-0.68	-0.26	0.04	1.50	0.18	0.42	-0.24	0.78	0.38	-0.22	0.31	46,XX
**D6**	0.66	-0.08	2.10	-0.66	0.16	0.65	-0.11	-0.83	-0.05	-2.11	-0.34	0.06	0.50	0.96	0.75	-0.40	-0.27	-0.61	0.10	-1.18	-0.45	-1.06	46,XY
**D7**	0.20	0.34	2.24	0.18	0.73	1.09	-0.56	-0.89	-0.72	-1.38	-0.05	0.92	-0.09	-0.63	0.22	-0.09	-0.50	-0.61	0.11	-0.59	-0.21	-1.98	46,XY
**D8**	-0.33	0.07	0.13	-1.14	0.92	-0.01	-0.10	-0.23	-0.44	-0.06	0.87	0.01	-0.04	-0.27	-0.48	0.18	-0.35	0.45	0.20	0.61	-0.27	0.28	46,XX
**D9**	0.28	-0.25	-0.18	-3.14	0.02	-0.26	-1.79	-1.31	-1.80	-0.09	1.00	0.82	-0.41	0.60	0.57	-0.30	1.21	-0.89	0.58	0.39	6.43	0.76	47,XX,+21
**D10**	-0.04	-0.09	0.86	0.04	1.42	0.63	0.24	0.37	1.27	-0.78	0.94	-0.56	-0.40	-0.50	0.48	-0.49	-0.72	0.28	-0.36	-0.53	-0.82	-0.15	46,XY
**D11**	0.04	0.20	-0.90	-1.49	0.20	0.55	-1.89	0.04	0.09	0.85	1.17	-1.24	-0.35	-0.28	0.45	0.13	0.78	0.65	-0.43	0.99	0.81	0.75	46,XX

Table S1. Aneuploidy test results of the between-sample comparison method (like Fan et al.) for all chromosomes using the same data preparation as the within-sample method (filtering and GC-correction). True positives are shown in green text, false positives are red, true negatives are shown in normal black font while false negatives were not found. Turquoise bold text marks results that are caused by a subchromosomal aberration thus their results can be interpreted both as false positives as it is not truly an aneuploidy, as well as true positives as they do correctly mark an aberrated chromosome. Samples with purple results have 69 chromosomes hence their results can not be interpreted as truly wrong or right as all chromosomes were expected to increase in relatively equal amounts.

Sample	Chr	Start	Stop	Mb	AvgZ	Details
A1	1	202	221	19	3.1157	46,XX
	5	30	58	28	-3.194	
	8	14	33	19	-3.215	
	16	37	54	17	-3.321	
A3						46,XY
A5	13	35	94	59	5.4563	47,XY,+13
A7						46,XY
A9	18	24	74	50	5.2281	47,XY,+18
A11	5	102	116	14	3.5458	47,XY,+21
	21	16	46	30	7.8657	
A13	18	19	60	41	4.6227	47,XY,+18
A15						46,XX
A17	21	16	46	30	10.464	47,XY,+21
A19						46,XY
A21						46,XX
A23						46,XY
A25	13	24	99	75	6.3457	47,XY,+13

Sample	Chr	Start	Stop	Mb	AvgZ	Details
C1						45,X[47]/46,XX[4]
C2						45,X[5]/46,XX[25]
C3						45,X/46,XX
C4						46,X,del(X)(p11.2)[23]/45,X[33]
C5	13	60	76	16	-3.233	46,XX,del(13)(q12.3q14.3)
C6						**46,XX,i(18)(q10)**
C7						46,XX,r(14)(p11.1q32)[26]
C8	4	106	120	14	3.1897	47,XXY
C9						47,XXY
C10						47,XXY
C12	22	26	38	12	3.9947	47,XY,+22
C13	12	48	59	11	3.6815	47,XY,+i(12)(p10)
	12	72	83	11	3.6044	
C14						47,XYY
C15						69,XXY
C16						69,XXY
C17						47,XXY
C18	22	23	46	23	5.2745	47,XY,+22
C19						**46,XY,7p+[8]/46,XY[12]**
C20						46,XY,t(4;10)(q27;p13)pat
C21						69,XXY/46,XX
C22						46,XY
C23						46,XY
C24						46,XY
C25						46,XY
C26						46,XX
C27						46,XX
C28						46,XX
C29						46,XX
C30						46,XX
C31						46,XX
C32	21	22	38	16	6.7125	47,XX,+21
C33	21	16	45	29	6.8928	47,XX,+21

Sample	Chr	Start	Stop	Mb	AvgZ	Details
D1						46,XX
D2	21	22	36	14	4.5088	47,XY,+21
D3	3	87	100	13	3.1691	47,XY,t(5;12)(q1?3;q24.1)+21
	21	16	46	30	7.6283	
D4	21	16	46	30	6.0141	47,XX,+21
D5						46,XX
D6						46,XY
D7						46,XY
D8						46,XX
D9	21	22	38	16	5.0634	47,XX,+21
D10						46,XY
D11	7	82	97	15	-3.932	46,XX

Table S2. Subchromosomal between-sample comparison (individual bin method version based on the between sample comparison method as developed by Fan et al.) results using 1Mb bin sizes and the same data preparation steps as the within-sample comparison method (filtering and GC-correction). True positives are marked green, false positives are marked red in the middle columns, false negatives are marked red in the Details column.

Demultiplexing

Variable	Default	Description
Mismatches	1	Total allowed mismatches in tag sequence when demultiplexing data.

Soap

Variable	Default	Description
v	0	Total allowed mismatches in one read, 0 = none.
r	0	How to report repeat hits, 0 = remove.

RETRO

Variable	Default	Description
W	4	Maximum base pairs distance between two adjacent reads to consider them part of a tower.
T	4	Minimum amount of reads within W distance of each other to consider it a tower and removing reads.

WISECONDOR

Variable	Default	Description
f	0.1	Smoothing span for LOWESS fit.
i	3	Iterations for LOWESS fit.
B	1Mb	Bin sizes used for testing and reference tables. Binned data is saved to separate files.
MaxRefBins	100	Maximum amount of reference bins for each target bin, improves runtime at barely any quality cost.
MinRefBins	10	Minimum amount of reference bins for each target bin to make calls.
T_D	MaxInt	Optional starting threshold for quality filtering of reference bins. High value for no effect.
T_A	2	Amount of cycles for reference bins quality cycles. Higher values result in higher quality requirements.
MaxRounds	5	Maximum amount of testing cycles before calling deviating bins.
w	11	Sliding window size, takes (w-1)/2 additional bins from each side of the tested bin for Stouffers z-score.
Robustness	1	Amount of bins to remove from both the top and bottom of the window when using Stouffers z-score.
MaxBinSkip	2	Maximum gap between two adjacent deviating areas to consider them part of a single aberration.
MinLength	10	Minimum size in bins of detected aberration before calling subchromosomal aberrations.
T^a	0.5	Threshold on ratio of deviating bins on a chromosome to call anoploidy.

Table S3. Overview of all variables used in the pipeline for WISECONDOR, subdivided per step in the script. As shown, numerous variables are required for classification and finding optimal values for them requires a lot of testing.

Sample	Cov	AvgASD	AvgGCc	Change
A1	0.42	**12.20**	**7.83**	-4.37
A3	0.44	**7.79**	**5.34**	-2.45
A5	0.72	3.98	4.37	**0.39**
A7	0.64	4.21	4.32	**0.11**
A9	0.35	4.76	4.76	0.00
A11	0.35	**5.81**	**5.16**	-0.65
A13	0.71	4.32	4.35	**0.03**
A15	0.71	**5.19**	3.79	-1.40
A17	0.82	4.70	4.61	-0.09
A19	0.71	3.57	3.52	-0.05
A21	0.41	4.48	4.18	-0.30
A23	0.49	4.87	4.12	-0.75
A25	0.86	**5.34**	4.53	-0.81

Sample	Cov	AvgASD	AvgGCc	Change
C1	1.06	3.64	3.77	**0.13**
C2	1.00	**5.34**	3.61	-1.73
C3	0.46	4.72	4.28	-0.44
C4	0.36	**5.33**	4.68	-0.65
C5	0.18	**7.54**	**5.93**	-1.61
C6	0.31	**5.75**	4.87	-0.88
C7	0.74	**7.18**	3.84	-3.34
C8	0.50	**9.65**	**6.94**	-2.71
C9	0.39	**7.07**	4.26	-2.81
C10	0.58	**7.71**	4.11	-3.60
C12	0.37	**6.58**	4.56	-2.02
C13	0.39	4.79	4.80	**0.01**
C14	0.47	**5.88**	4.24	-1.64
C15	0.71	**9.57**	4.31	-5.26
C16	0.32	**8.09**	4.63	-3.46
C17	0.64	4.60	4.76	**0.16**
C18	0.41	**6.52**	4.53	-1.99
C19	0.23	**6.64**	4.98	-1.66
C20	0.39	4.93	4.51	-0.42
C21	0.60	**11.53**	4.96	-6.57
C22	0.06	**6.08**	**6.23**	**0.15**
C23	0.06	**6.86**	**6.34**	-0.52
C24	0.04	**8.11**	**6.96**	-1.15
C25	0.06	**7.21**	**6.32**	-0.89
C26	0.23	4.17	4.43	**0.26**
C27	0.35	4.37	4.05	-0.32
C28	0.22	4.28	4.51	**0.23**
C29	0.16	**5.09**	**5.12**	**0.03**
C30	0.23	4.53	4.80	**0.27**
C31	0.22	**5.96**	4.70	-1.26
C32	0.15	**6.02**	**6.26**	**0.24**
C33	0.36	**6.39**	4.81	-1.58

Sample	Cov	AvgASD	AvgGCc	Change
D1	0.60	4.97	4.21	-0.76
D2	0.50	**15.93**	4.85	-11.08
D3	0.57	4.62	4.67	**0.05**
D4	0.55	4.83	4.33	-0.50
D5	0.55	4.66	4.08	-0.58
D6	0.63	**16.56**	4.47	-12.09
D7	0.63	4.44	4.13	-0.31
D8	0.42	4.49	4.24	-0.25
D9	0.56	4.92	4.44	-0.48
D10	0.50	**8.42**	4.34	-4.08
D11	0.35	**7.43**	**5.24**	-2.19

Table S4. Results of applying the LOWESS GC-correction to the data. The percentual average standard deviation per bin before correction is shown per sample in the AvgASD column, the results after GC-correction are shown in the AvgGCc columns, all values over 5 are shown in bold as we consider 5% a threshold for stable calls, based on the assumed minimum of 10% fetal DNA in the samples. The last column shows the changes in these values, a bold font marks samples where the GC-correction increased this value.

	1	2	3	4	5	6	7	8	9	10	11	12	13	14	15	16	17	18	19	20	21	22	Karyotype
**A1**	**3.25**	1.37	0.87	-0.21	-0.97	1.51	-0.24	-1.05	-1.38	0.18	-0.71	-0.33	0.08	-0.26	1.19	-2.70	1.12	-0.78	**-4.56**	-2.44	0.42	0.45	46,XX
**A3**	0.95	1.60	-0.76	-0.18	-1.33	0.49	-0.42	-1.42	0.09	0.72	-2.49	0.46	1.03	-0.38	2.61	-2.25	0.86	-0.52	**-5.55**	0.79	2.37	0.25	46,XY
**A5**	-1.23	-1.30	-0.43	-0.64	-0.08	-0.79	-1.09	0.06	0.49	-1.42	0.38	-0.72	**22.22**	-1.14	-0.36	1.12	-1.32	-1.16	-1.12	1.28	1.17	0.35	47,XY,+13
**A7**	0.07	0.25	0.99	-1.32	1.24	0.14	-0.08	1.12	0.40	1.56	0.20	-0.78	-0.38	-0.36	0.49	0.99	-1.47	1.36	-0.86	0.66	-0.12	0.36	46,XY
**A9**	0.03	-0.47	-1.10	-1.77	-1.43	-1.03	-0.20	-1.34	-1.29	0.98	-0.56	-0.69	0.09	-0.93	-0.04	-0.53	-0.36	**16.36**	-0.36	1.48	0.32	-0.56	47,XY,+18
**A11**	0.94	0.87	-1.20	0.11	2.18	-0.44	0.76	-0.56	-0.93	-2.01	2.59	0.01	-0.52	-1.79	0.29	-2.29	0.65	-1.31	-0.02	0.79	**20.53**	-0.31	47,XY,+21
**A13**	0.70	-1.25	1.13	-0.74	-0.83	-1.21	-1.93	-2.17	0.17	-0.46	-0.24	-0.74	0.83	-0.71	0.92	-2.34	-1.56	**14.27**	-1.84	-0.03	-0.29	1.11	47,XY,+18
**A15**	-1.68	-2.30	2.01	-0.05	0.95	-1.81	0.05	1.30	0.19	-1.83	1.42	-0.49	1.48	-1.20	-0.23	0.68	-0.74	1.37	0.38	2.21	2.52	-0.20	46,XX
**A17**	-0.69	-1.03	1.47	0.40	1.16	-1.59	-1.66	0.34	1.55	0.48	0.13	0.34	0.33	0.57	-0.16	-0.62	-1.81	0.47	-1.76	-0.13	**35.56**	-1.42	47,XY,+21
**A19**	0.71	-1.07	1.20	-1.07	0.36	0.72	-0.02	-0.58	0.41	-0.72	2.43	-1.46	**3.06**	1.41	0.01	-1.64	-2.53	0.02	-0.73	0.87	-0.18	0.17	46,XY
**A21**	0.20	-0.24	0.47	-0.46	0.60	-1.46	0.54	-1.18	0.96	-1.54	0.90	-0.69	0.37	-1.11	1.94	0.01	-0.57	2.10	0.21	2.25	0.49	2.16	46,XX
**A23**	-0.69	0.07	0.51	1.81	1.59	-0.22	0.83	1.19	0.97	-0.20	-0.61	2.48	-1.77	-0.81	**-3.56**	1.22	-2.48	-1.18	-0.33	-0.18	-0.30	-0.75	46,XY
**A25**	-1.25	-1.44	0.40	-1.41	-0.05	-1.16	-0.92	-0.71	0.32	-0.85	-0.16	0.04	**29.30**	0.65	0.63	1.32	-1.08	-0.04	-0.37	-0.57	0.35	0.27	47,XY,+13

	1	2	3	4	5	6	7	8	9	10	11	12	13	14	15	16	17	18	19	20	21	22	Karyotype
**C1**	1.49	-2.12	-0.47	0.21	-1.49	-2.69	-1.46	-0.36	0.97	0.16	1.08	-0.33	-0.21	-0.28	0.31	2.45	1.81	1.88	-0.19	2.54	-0.73	1.45	45,X[47]/46,XX[4]
**C2**	1.88	-1.81	-0.28	-2.08	0.55	-1.65	-1.99	-0.33	0.72	-0.14	0.98	-0.03	-0.49	-0.16	1.12	**3.66**	0.37	-0.76	**4.20**	**3.07**	-0.20	-0.24	45,X[5]/46,XX[25]
**C3**	-0.28	-1.11	0.40	-0.20	-1.54	-1.80	0.34	1.06	0.43	-0.14	-0.75	-0.29	0.26	0.33	0.19	1.54	0.17	0.54	-0.41	0.96	-0.34	0.36	45,X/46,XX
**C4**	-0.92	0.81	-1.17	-2.45	0.04	0.33	0.20	1.91	0.60	-1.21	1.92	1.07	0.22	**3.84**	-1.09	0.90	-1.08	-0.49	1.52	0.80	0.33	-0.24	46,X,del(X)(p11.2)[23]/45,X[33]
**C5**	1.40	-0.64	0.24	-0.28	-0.61	1.45	-0.30	0.38	-0.10	1.82	0.30	0.62	**-6.72**	-0.91	0.00	2.51	0.35	0.05	0.55	0.64	-0.64	-0.30	46,XX,del(13)(q12.3q14.3)
**C6**	0.39	-1.34	-0.07	-0.86	-2.39	-0.20	-1.49	-0.95	-0.53	-2.85	-0.88	0.58	0.92	-0.76	0.10	0.61	0.40	**5.60**	0.87	1.83	-0.17	1.17	46,XX,i(18)(q10)
**C7**	-1.24	-0.26	-0.43	0.63	-0.87	-2.32	0.20	0.22	0.21	0.57	1.53	-1.34	0.33	-0.86	0.90	1.27	0.60	-0.41	1.80	-0.04	0.31	0.76	46,XX,r(14)(p11.1q32)[26]
**C8**	**-3.58**	-0.70	-0.56	**3.88**	2.03	1.35	1.67	2.81	-0.74	-0.56	-2.22	2.26	-2.31	-0.27	-2.63	0.96	-2.90	1.07	-0.78	**-3.12**	-1.87	1.40	47,XXY
**C9**	0.67	0.30	0.83	0.62	-0.32	-0.89	0.12	-1.03	-1.79	0.19	-0.48	-0.77	1.20	0.12	-0.34	-0.53	0.54	-0.73	-0.01	0.74	-0.70	-0.37	47,XXY
**C10**	2.89	0.29	2.09	2.38	-0.07	-1.38	1.02	0.74	-0.60	-1.96	-1.53	-1.11	1.33	-1.39	-1.52	0.72	0.44	-0.22	1.55	0.16	-0.64	0.41	47,XXY
**C12**	-0.34	-1.30	0.04	-1.18	0.80	-0.78	-1.29	0.74	0.40	0.77	0.17	0.03	1.21	1.43	0.12	-0.77	0.74	-0.72	-0.81	1.05	2.16	**10.20**	47,XY,+22
**C13**	-0.01	-0.33	-1.61	-1.28	-1.14	-1.69	-0.91	0.23	-0.43	0.46	1.50	**9.43**	0.65	-1.31	1.18	0.39	0.58	0.25	0.07	1.97	-0.33	0.62	47,XY,+i(12)(p10)
**C14**	-0.42	-1.60	0.74	1.55	-0.50	-0.78	-1.01	1.11	-0.06	-1.07	-0.07	-0.06	1.84	-0.76	-0.88	1.21	-0.94	2.47	-1.66	0.39	0.03	-0.30	47,XYY
**C15**	2.55	0.07	1.41	-2.90	0.77	-0.90	-0.40	-0.52	0.09	-1.43	2.34	0.70	-1.47	-1.58	-0.65	1.47	-0.54	-0.61	1.12	1.46	0.04	0.02	69,XXY
**C16**	-1.48	1.16	1.71	-0.07	0.30	-1.21	-0.50	0.95	2.76	-1.37	-0.77	-1.32	-1.05	-0.26	-0.32	-0.43	-1.28	-0.73	0.44	1.38	0.26	-2.29	69,XXY
**C17**	-0.02	0.00	1.42	-1.82	0.60	0.85	1.63	0.67	0.82	0.42	1.68	-0.23	-0.95	-0.01	-1.84	1.43	1.06	-0.60	2.62	2.11	-1.14	-1.69	47,XXY
**C18**	-1.06	-0.90	1.52	0.24	-1.77	0.01	1.60	0.10	-0.33	0.62	-1.31	-0.52	-0.80	-1.46	-0.06	1.55	-1.57	0.85	-0.13	1.18	1.58	**17.73**	47,XY,+22
**C19**	-0.47	-0.81	-1.18	-0.04	0.34	-0.56	-0.67	0.10	-0.57	-0.50	1.28	0.51	2.02	0.12	-0.83	-0.81	1.07	-0.01	1.42	1.74	1.41	-1.23	46,XY,7p+[8]/46,XY[12]
**C20**	-0.51	-0.43	-1.67	-0.95	-0.40	-0.50	-0.79	0.97	1.48	2.55	-0.29	-0.24	-0.11	0.46	1.07	2.19	2.39	0.10	1.02	2.49	1.92	0.65	46,XY,t(4;10)(q27;p13)pat
**C21**	2.40	-1.36	0.00	-2.55	0.74	-0.82	-1.86	1.12	0.35	-1.92	2.79	0.46	-0.57	-0.37	-1.06	2.93	1.56	-0.92	**6.01**	**3.74**	-1.86	0.47	69,XXY/46,XX
**C22**	0.12	-0.67	-0.64	-0.35	-0.39	0.78	0.56	0.68	0.35	0.66	-0.18	-1.65	2.04	-1.28	0.13	-0.09	-0.72	0.67	-0.65	0.54	-0.29	-1.42	46,XY
**C23**	0.23	-0.10	-0.41	0.16	-0.37	-0.37	-1.27	0.41	-0.32	0.67	0.21	-1.16	-0.67	-0.10	-0.18	0.87	-0.17	-0.68	-0.09	1.12	-0.59	-0.55	46,XY
**C24**	-0.40	0.09	-0.73	1.18	-0.31	-0.03	0.56	-0.26	1.92	-0.24	1.01	0.25	0.18	1.10	-0.85	0.67	0.40	-1.07	0.44	-0.72	-0.63	-0.46	46,XY
**C25**	1.12	1.03	-0.31	-0.15	-1.46	-1.03	-1.31	-0.24	0.73	0.63	-0.09	0.63	-2.12	-0.84	-0.27	-1.23	0.17	0.76	0.57	1.30	-1.38	1.67	46,XY
**C26**	-1.30	-0.20	-0.30	-0.93	1.05	1.08	0.01	0.33	0.86	-0.89	0.23	0.67	-0.59	-0.78	-1.02	2.67	-0.32	-1.16	1.19	0.55	0.44	-2.13	46,XX
**C27**	0.29	0.06	0.15	0.25	-0.96	0.38	-0.45	0.59	-0.60	0.34	-0.05	0.79	0.30	-0.53	0.61	1.17	0.52	0.93	1.94	0.62	-0.19	0.01	46,XX
**C28**	-0.15	0.65	-0.46	-2.62	-0.92	-1.55	-0.40	0.06	1.38	0.22	1.40	1.29	-0.29	-1.13	0.78	0.04	0.77	-0.52	1.87	0.12	-0.50	0.34	46,XX
**C29**	1.90	-0.34	-0.24	-1.69	-0.01	-1.94	-0.41	0.27	-1.33	0.61	-0.80	0.70	0.40	-0.43	1.24	2.77	0.88	-1.03	1.53	0.19	0.33	0.65	46,XX
**C30**	-1.99	-1.32	-0.52	-0.84	-1.22	-1.35	-1.30	-0.94	-1.37	0.90	-0.44	-1.62	-0.44	**6.63**	0.12	2.59	1.35	2.36	0.82	1.29	1.07	0.20	46,XX
**C31**	-1.36	-0.61	0.26	0.68	1.52	0.01	1.18	1.05	-1.83	-0.60	1.08	0.02	0.47	-0.55	-0.52	0.35	0.07	2.54	1.04	0.26	-0.95	-0.77	46,XX
**C32**	-0.80	1.08	-0.95	-1.53	-0.16	-1.36	-1.19	-0.03	-1.30	-1.00	0.80	2.28	-1.75	0.20	1.58	-0.58	0.03	-1.02	-0.60	1.42	**11.63**	0.30	47,XX,+21
**C33**	-0.95	0.89	-1.23	-1.69	-0.06	0.90	-0.80	0.65	-0.61	0.80	0.61	0.00	-0.39	-2.11	0.26	2.13	0.14	0.30	2.30	0.86	**19.63**	-0.97	47,XX,+21

	1	2	3	4	5	6	7	8	9	10	11	12	13	14	15	16	17	18	19	20	21	22	Karyotype
**D1**	1.16	0.16	**3.49**	-0.14	0.37	0.62	-2.60	-1.55	0.13	1.54	2.77	-0.17	1.06	-1.10	-0.86	-2.56	-1.34	-0.62	-1.63	0.92	-1.45	-1.04	46,XX
**D2**	0.61	0.42	-0.03	-1.64	0.66	0.00	-2.50	0.24	-0.15	0.07	2.02	-0.71	0.17	-0.99	-1.39	1.86	0.70	-2.22	2.45	2.10	**9.43**	0.83	47,XY,+21
**D3**	2.45	0.14	2.95	-0.80	-0.54	0.08	-2.20	-0.49	-0.44	-0.08	0.35	0.41	-1.94	-1.48	-0.49	-0.61	1.10	-2.70	-1.96	-1.44	**22.41**	-0.48	47,XY,t(5;12)(q1?3;q24.1)+21
**D4**	-0.91	0.15	-0.33	-1.46	0.14	-1.63	-0.54	-0.38	0.74	0.99	0.57	0.96	-1.53	0.90	-0.49	0.81	1.21	-1.03	1.64	2.76	**19.93**	1.52	47,XX,+21
**D5**	-0.08	-1.41	2.58	-0.12	0.64	-1.71	-1.94	0.37	0.41	-1.19	0.06	-1.20	-0.64	0.61	0.86	1.98	1.75	-0.30	2.53	1.01	0.07	0.82	46,XX
**D6**	**3.17**	-1.09	1.12	-2.44	0.02	1.24	-0.86	-1.68	0.55	-1.69	1.28	0.16	1.12	0.80	1.17	0.51	2.03	-0.96	1.10	0.38	-0.55	0.77	46,XY
**D7**	1.33	0.99	2.68	-1.32	1.65	1.82	-1.59	-1.13	-0.25	-2.03	0.84	0.76	-0.94	-0.88	0.61	-0.82	-0.01	-0.41	0.06	1.11	-0.34	-2.40	46,XY
**D8**	-0.66	0.32	0.32	-1.55	1.85	-0.38	0.03	-0.03	-0.71	1.14	1.17	0.35	0.31	-1.02	-0.24	-0.89	0.46	0.58	0.74	1.76	-0.29	0.17	46,XX
**D9**	2.30	0.00	0.96	**-5.11**	-0.26	-1.21	**-3.62**	-1.58	-1.48	0.99	2.37	1.70	-0.57	0.33	2.78	-0.68	2.24	-1.41	1.12	1.80	**12.36**	1.54	47,XX,+21
**D10**	-0.24	-0.72	0.56	-0.18	2.36	1.19	0.23	0.98	1.56	-0.55	0.94	-0.61	-0.64	-0.74	-0.72	-0.77	-0.27	0.22	-2.18	-0.92	-1.62	-0.40	46,XY
**D11**	0.01	1.03	-1.43	-1.11	0.55	1.56	-1.88	0.51	0.42	2.34	1.55	-0.65	0.05	0.31	0.79	0.22	2.51	0.53	0.89	1.33	1.24	0.69	46,XX

Table S5. Overview of test results when applying a chromosome wide Stouffer's z-score when using the within-sample comparison method. To increase robustness the top and bottom 5% of the z-scores for each chromosome were ignored. All values deviating over 3 are shown in bold text. True positives are marked in green, false positives in red, true negatives are black and false negatives were not found. Values shown in turquoise were cases where the chromosome was not an aneuploidy but the sample did have an aberration on that chromosome. Although this approach resulted in many false positives, chromsomes 13, 18, 21 and 22 were always identified correctly. Application of this script to identify aneuploidy cases for these chromosomes appears possible without further alterations. We decided to look into alternative methods as we also wanted to call subchromosomal aberrations as well as other aneuploidies.
